# Suspension-associated dislocation of the jaw in hanging

**DOI:** 10.1007/s00414-023-03059-1

**Published:** 2023-07-18

**Authors:** Joanna Glengarry, Megane Beaugeois, Lyndal Bugeja, Richard Huggins, Chris O’Donnell

**Affiliations:** 1 Forensic Services and Department of Forensic Medicine, Victorian Institute of Forensic Medicine and Monash University, 65 Kavanagh Street, Southbank, VIC Australia; 2 Department of Forensic Medicine, Monash University, 65 Kavanagh Street, Southbank, VIC Australia; 3 Oral and Maxillofacial Surgery Department, Austin Health, 145 Studley Road, Heidelberg, VIC Australia

**Keywords:** Hanging, Neck compression, Temporomandibular joint, Temporomandibular dislocation, Post-mortem computed tomography (PMCT), Autopsy

## Abstract

Hanging is a common type of death, and the role of the medical investigation of such deaths by a forensic pathologist not only requires the determination of the cause of death but providing information to assist in the determination of the manner of death. The forensic pathologist should be well versed in the spectrum of injuries known to be associated with neck compression, to document injuries known to be associated with hanging, but also to identify those that are inconsistent with self-inflicted hanging or that may suggest the involvement of a third party in the death. Comprehensive identification and correct interpretation of external and internal injury are crucial for the appropriate degree of police and coroner/medical examiner investigation. We present two cases of deaths believed to be caused by self-inflicted hanging that were observed to have unexpected unilateral dislocation of the temporomandibular joint identified on routine post-mortem computed tomography, without any evidence of involvement of a third party. This injury was unexplained and had not been previously observed at our Forensic Institute nor was it identified after a review of the published biomedical research literature. Issues regarding the cause of this abnormality, possible mechanisms, and the medicolegal significance of this finding will be discussed.

## Introduction

Hanging is a common type of death presented to forensic pathologists in countries with requirements that unnatural and unexpected deaths be independently investigated [1]. In Australia, the most recent cause of death statistics showed that among the 1937 deaths classified as hanging and strangulation, 98.5% (*n* = 1909) were determined as intentional self-harm, 0.7% (*n* = 13) were unintentional, 0.6% (*n* = 11) were assault, and 0.2% (*n* = 4) were undetermined^1^ [2].

The role of the medical investigation of such deaths by a forensic pathologist not only requires determination of the cause of death, but to provide information to assist the relevant authority in the determination of the manner of death (be it the medical examiner themselves or the coroner). This requires consideration to be given to the circumstances of death and for the medicolegal death investigation to rule out any other competing cause of death which may suggest a manner other than suicide. Therefore, in cases of death from hanging, one looks to document not only injuries that are known to be associated with hanging (such as an upsloping ligature abrasion to the neck), but also to identify those that may be inconsistent with self-inflicted hanging or that may suggest the involvement of a third party in the death. Consequently, the forensic pathologist must be cognizant of the spectrum of injuries that are known to be associated with death from hanging, so that cases with atypical injuries may be appropriately escalated for police investigation. Conversely, it is vital that injuries are not incorrectly attributed as being due to assault and creating unnecessary investigational work for police and unwarranted distress for the deceased’s next of kin.

We present two cases of deaths reported to the coroner that were believed to be caused by self-inflicted hanging and that were observed to have unexpected unilateral dislocation of the temporomandibular joint (TMJ) identified on routine post-mortem computed tomography (PMCT), without any suspicious circumstances surrounding the deaths. This injury was unexplained and had not been previously observed at our Forensic Institute nor was it identified after a review of the scientific research literature using one biomedical database (Medline via Ovid).

## Case report

### Preamble

Approximately 7000 deaths per year are reported to the coroner in our jurisdiction, with reportable cases being deaths that are unexpected; unnatural, following a medical procedure; or occurring in those in care or custody. Medicolegal death investigation initially comprises a preliminary review by a forensic pathologist of scene images, the circumstances as known at that time, examination of a full-body PMCT scan, and a comprehensive external assessment of the deceased. The wishes of the next of kin with regard to autopsy and any concerns they may have surrounding the death are determined. This informs the coroner’s decision on whether or not to direct a full autopsy examination. In the two cases presented here, a full autopsy examination was not performed. It is beyond the remit of this case report to discuss the complexities of the decision regarding the scope of the post-mortem examination in cases such as this, and there is available literature on this topic elsewhere [3, 4].

All individuals admitted to our institute undergo a whole-body PMCT scan using a mortuary-located dual-source SOMATOM Definition Flash CT scanner (Siemens Healthcare, Erlangen, Germany). Images were viewed using syngo.via version VB60A software (Siemens Healthcare, Erlangen, Germany). CT technique at our institution includes a head-to-toe scan range, at 120 kVp, 280 effective mAs, 1.5 mm slice thickness, pitch of 0.6, rotation time of 0.55 s, 500 mm field of view, and reconstruction kernel of B30f medium smooth.

### Case 1

**Fig. 1:**
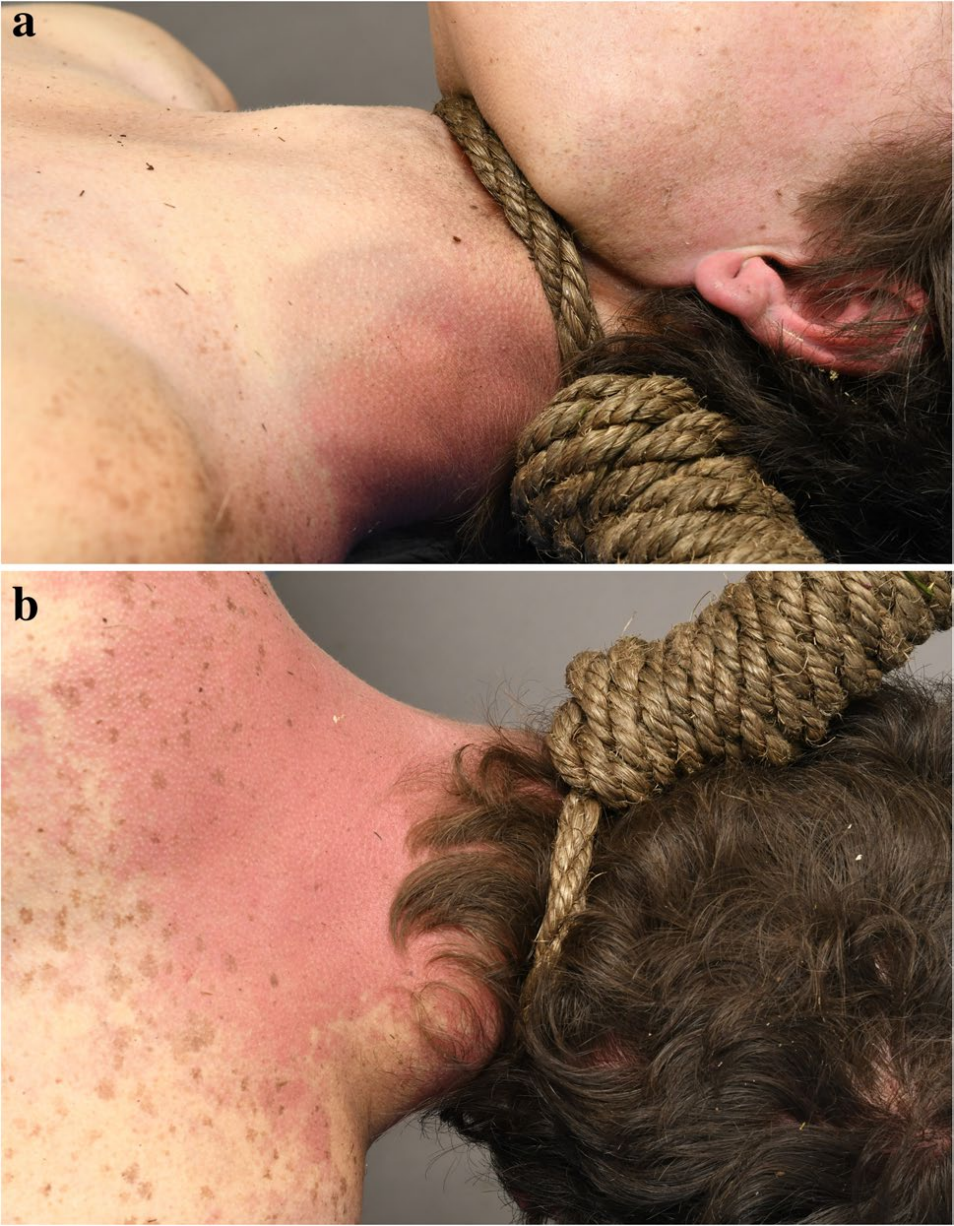
**a** Left lateral view, **b** posterior view of case 1 ligature, being a woven rope ligature fashioned as a “hangman-style” noose with a suspension point behind the left ear (left posterior)

A 67-kg, 173-cm-tall male aged in his twenties was located with a rope noose encircling his neck, fully suspended from a tree. He had a history of depression and recent interpersonal life stressors. The ligature (Fig. 1) was a 1.5-cm diameter woven rope fashioned as a “hangman-style” noose with a suspension point behind the left ear (left posterior).

**Fig. 2:**
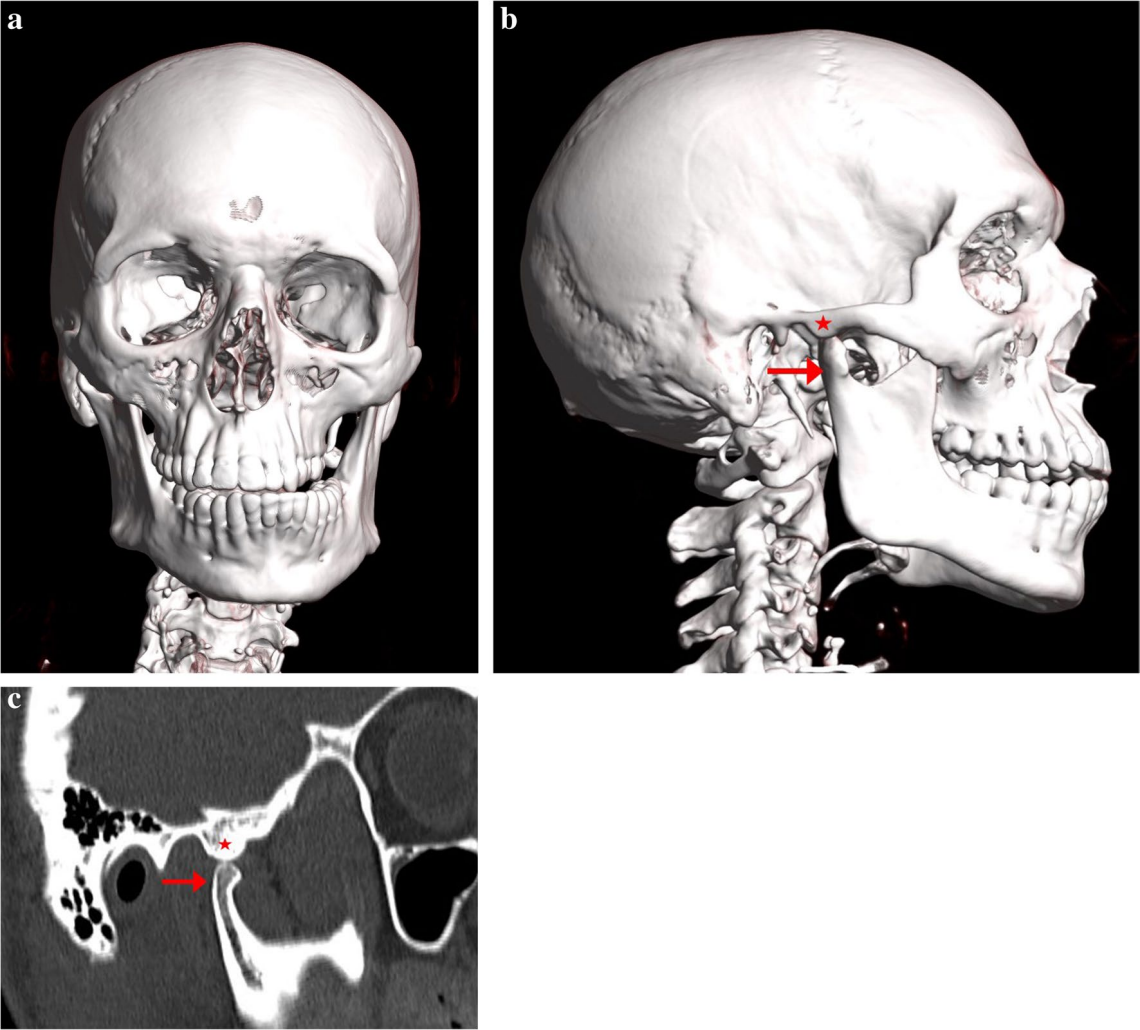
Case 1 three-dimensional reformat of the PMCT head: **a** anterior view showing right-to-left deviation of the mandible with a left crossbite, **b** right lateral view, and **c** sagittal image showing right mandibular condylar head displacement (arrow) anterior to the articular eminence (star)

Examination of the post-mortem CT scan showed a closed mouth with isolated right mandibular condylar head displacement anterior to the articular eminence, resulting in a left crossbite and left mandibular excursion (Fig. 2). The left condylar head position was congruous with the condylar fossa, as expected in a mouth-closed position. A fracture was present in the left greater cornu hyoid, and there was plastic deformation of the right greater cornu.

**Fig. 3:**
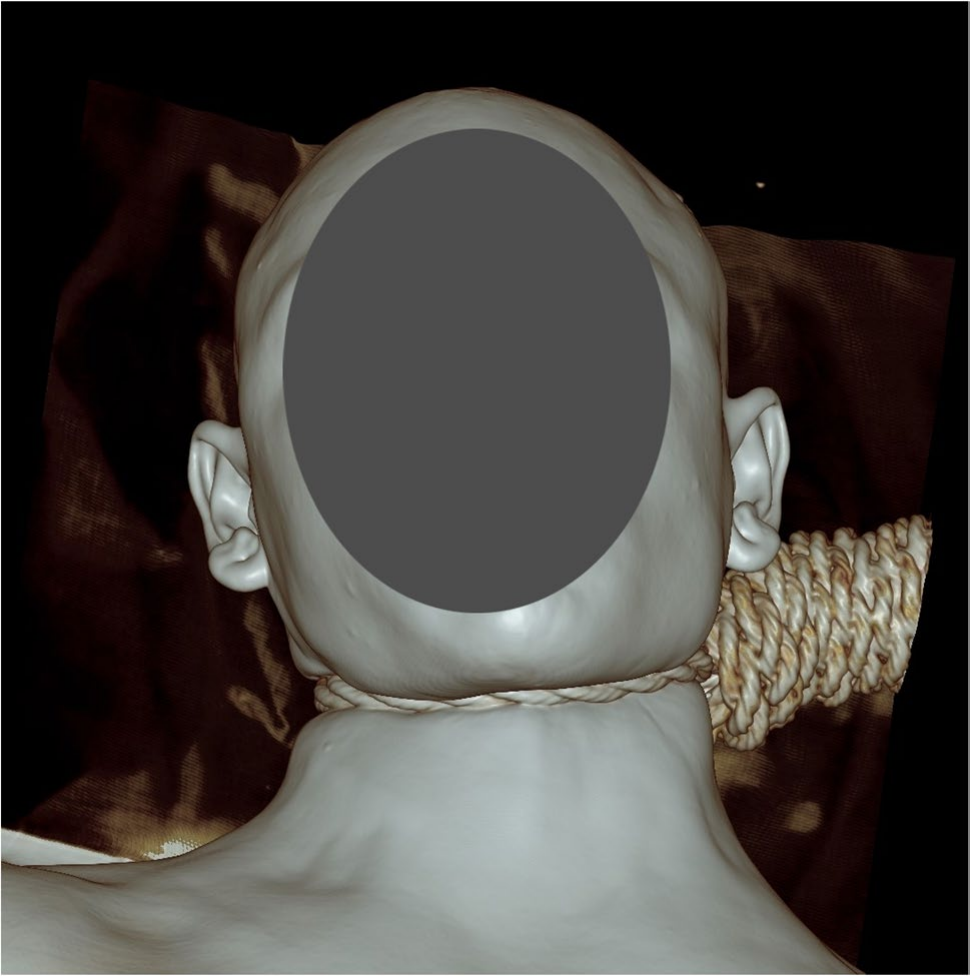
Case 1 three-dimensional reformat of the PMCT head showing the external appearance of the jaw, with deviation to the left. The rope ligature is in situ with a left posterior suspension point

External examination showed an adult male with established rigor mortis, fixed hypostasis, and no signs of decomposition. There was a deviation of the jaw toward the left (Fig. 3). The neck skin had a moderately deep ligature abrasion that comprised a dried, yellow–brown furrow with diagonal indentations within it in keeping with the ligature. Its lowest point was the right anterior neck, and it was upsloping across the neck, above the laryngeal prominence anteriorly, fading into the hairline behind the ears and with sparing of the posterior neck. The suspension point was behind the left ear. There were no other injuries to the neck or elsewhere on the body. There were no ocular or facial petechiae.

### Case 2

A 111-kg, 181-cm-tall male aged in his thirties was located with a rope noose encircling his neck, partially suspended in a factory. He had a history of recent interpersonal life stressors. The ligature was a 1.0-cm diameter braided rope that was described as encircling the neck, but the details of the configuration were not provided, and the ligature was removed at the scene by first responders.

**Fig. 4:**
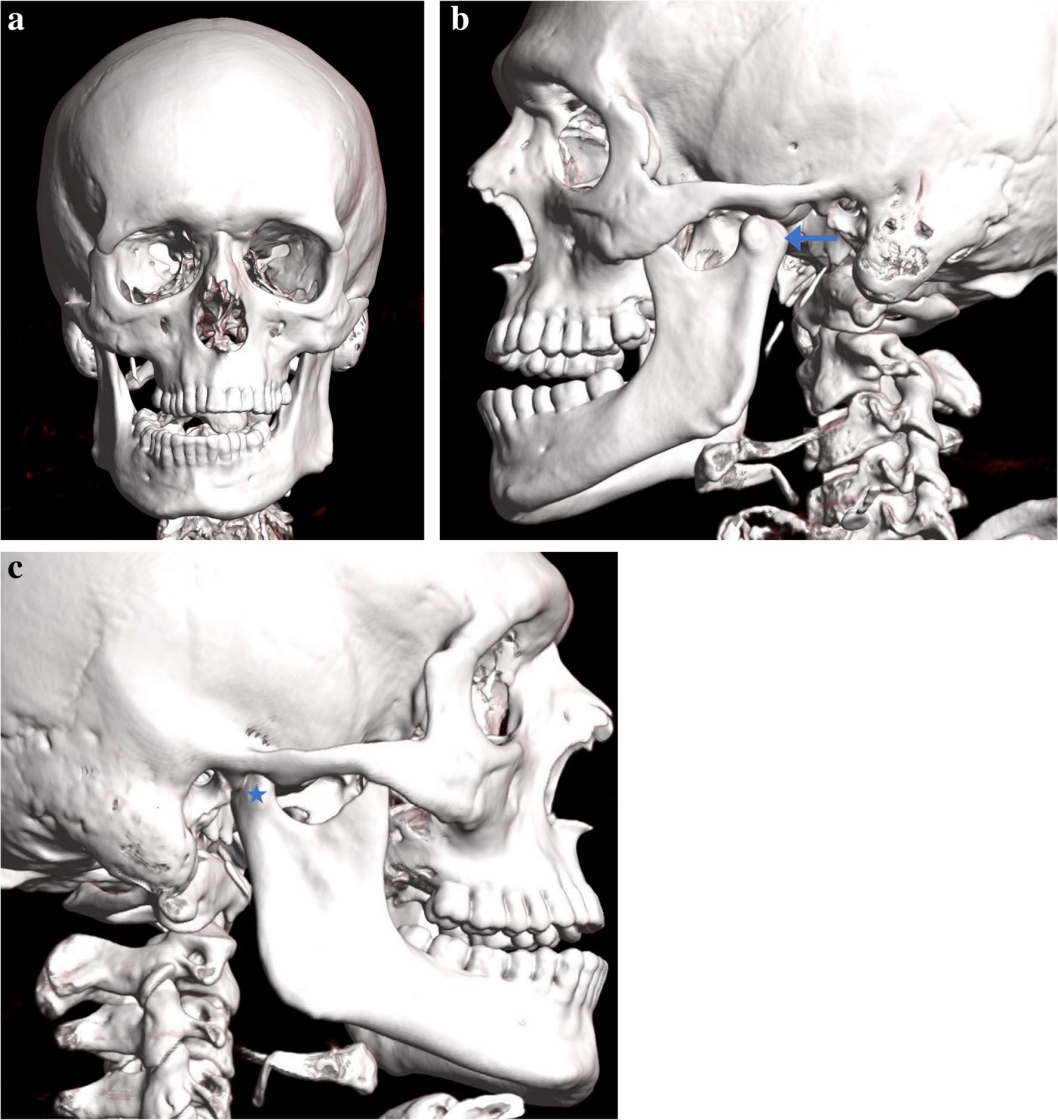
Case 2 three-dimensional reformat of the PMCT head: **a** anterior view showing a right crossbite, **b** left lateral view showing a near-closed mouth with left mandibular condylar head displacement anterior to the articular eminence (arrow), **c** right lateral view showing a right condylar head congruous with the condylar fossa (star)

Examination of the post-mortem CT scan (Fig. 4) showed a near-closed mouth with isolated left mandibular condylar head displacement anterior to the articular eminence, resulting in a right crossbite and right mandibular excursion. The right condylar head position was congruous with the condylar fossa, as expected in a mouth-closed position. There were right hyoid and superior cornu thyroid cartilage fractures.

**Fig. 5:**
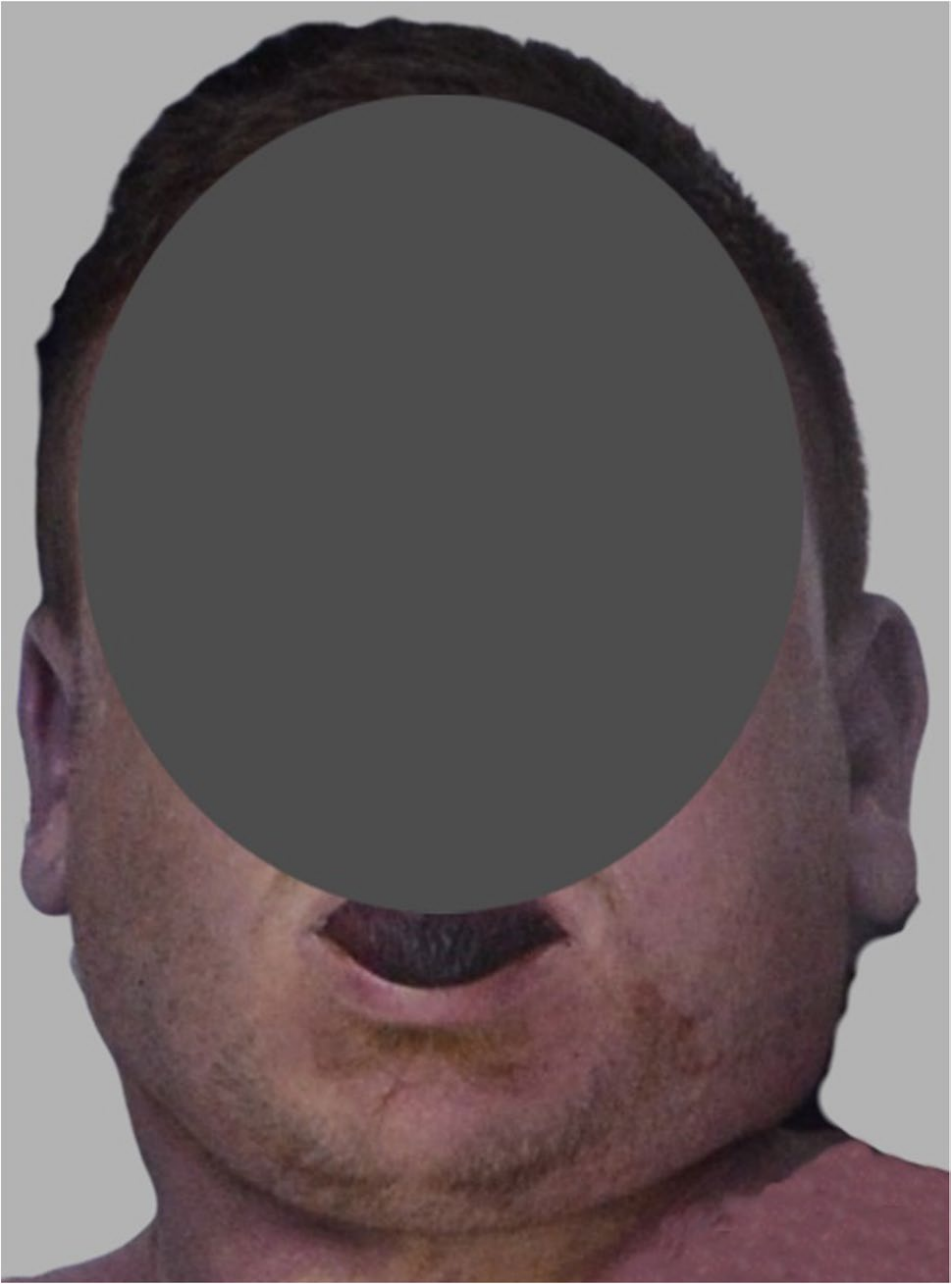
Case 2 with a subtle deviation of the jaw toward the right side

**Fig. 6:**
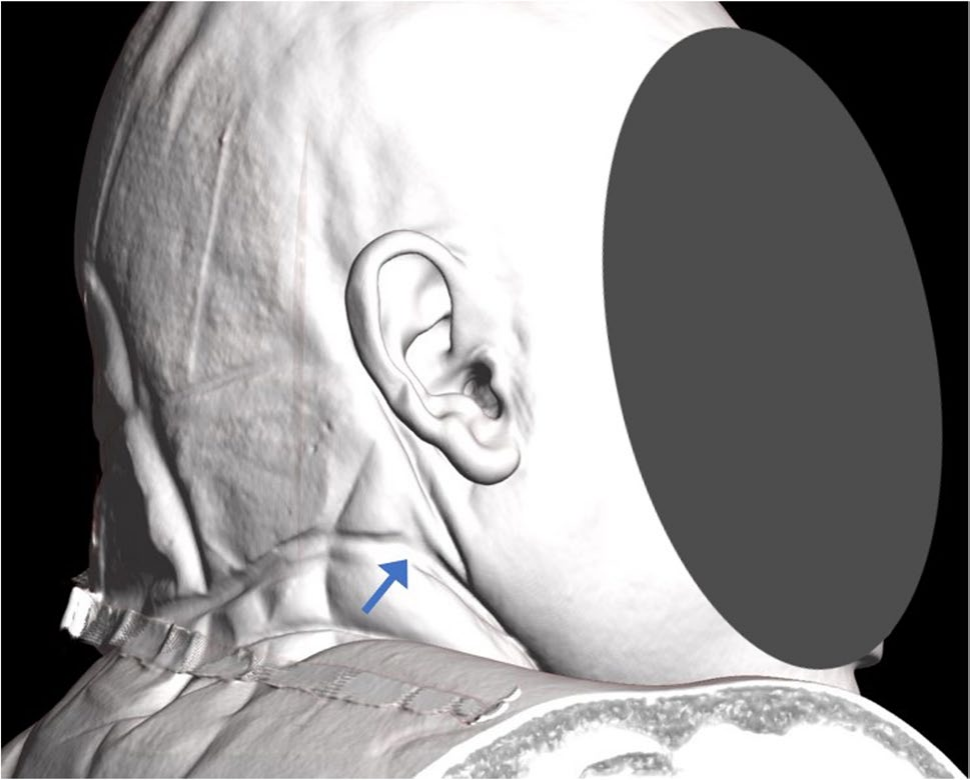
Case 2 three-dimensional reformat of the PMCT head demonstrating the ligature furrow with the likely suspension point behind the right ear (arrow)

External examination showed an adult male with established rigor mortis, fixed hypostasis, and no signs of decomposition. There was a subtle deviation of the jaw toward the right (Fig. 5). The neck skin had a dried, brown ligature abrasion in keeping with the ligature. Its lowest point was the left anterior neck, above the laryngeal prominence anteriorly and diagonally upsloping across the left and right sides of the neck (steeper on the right than the left). It traversed the posterior neck just below the hairline posteriorly (higher on the right than the left). The proposed suspension point was behind the right ear (Fig. 6), just below the hairline (right posterior). There were scattered conjunctival and scleral petechiae. There were no other injuries to the neck or elsewhere on the body.

## Discussion

It is well recognized that hanging causes a range of injuries primarily to the head and structures of the neck, the most typical of which are an upsloping ligature abrasion of the neck skin, cutaneous and ocular petechiae, fractures of the hyoid bone, laryngeal cartilages, and cervical spine [5]. Injuries to the limbs are also well recognized in these deaths [6]. When presented with a death purporting to be self-inflicted hanging, it is the remit of the forensic pathologist to assess the deceased’s injuries to not only assist in independently confirming this proposition, but to critically evaluate the finding of any injury that may suggest otherwise.

We have described two cases of unilateral TMJ dislocation (TMJD) in cases of lethal hanging. To our knowledge, this is not a previously recognized finding in such deaths and, as such, prompts consideration to the issues of TMJD causation, significance, and why this entity has not been previously identified. The cause of the TMJD is of the utmost importance in assessing the significance of this finding. A range of possibilities exist, including artifact, antemortem trauma, or ligature-related dislocation, and clearly, the implications of each vary.

Post-mortem artifacts as mimics of injury are commonly encountered [7], and forensic pathologists should be familiar with the decomposition of human remains and injury mimics that may arise as a consequence of decomposition. Well-recognized artifactual hemorrhages have been described in the neck which may cause consternation in hanging deaths if not correctly recognized [8, 9]. As decomposition progresses, disarticulation of joints may occur [10], which includes the TMJ. The two cases presented showed only early decompositional changes, not to the extent that skeletal changes would be expected, and this hypothesis would appear unlikely in these cases. It is known that the condylar heads of the mandible normally translate forwards from the condylar fossa in an open-mouth position [11], but many hanging deaths have a closed-mouth position (as in the cases presented), and unilateral TMJD is not in keeping with this.

The discovery of unexpected injury raises the specter of antemortem blunt force trauma or even the involvement of another in the death. In these two cases, the decedents had either diagnosed depression or an increase in life stressors as a potential trigger for suicide. A thorough police investigation disclosed no history of recent assault and found no suspicious features in the scene or circumstances. The external examination of the body did not reveal facial bruising or injuries elsewhere to suggest an assault. Notwithstanding that the distinction between homicidal and self-inflicted hanging may be problematic [12], there was no suggestion of antemortem trauma, and no mechanism of injury relating to body handling was identified in either case.

It therefore seems possible that TMJD represents a previously unrecognized artifact of ligature suspension. In our two cases, the TMJD occurred on the side opposite the point of ligature suspension. A postulated mechanism is that the mandibular head on the side of suspension is pulled up into the mandibular fossa, fixing it in position, but allowing distraction of the contralateral mandibular head out of the fossa by the pull of the ligature opposite the suspension point, triggering the unilateral dislocation. It is unknown if the decedents had an element of pre-existing TMJ dysfunction, increasing their vulnerability to TMJD. Dislocation may be assisted by muscle relaxation in the post-mortem period. The TMJD persisted even after removal of the ligature, but rigor mortis was fully fixed in both men (including the pterygoid and masseter muscles) which may have impacted the ability of the condyle to relocate.

To our knowledge, the finding of TMJD in deaths from hanging has not been previously recognized or reported. While injuries are typically identified and documented during a full autopsy examination, the TMJ region is not routinely examined, and this occurs for a combination of reasons. Facial asymmetry due to unilateral TMJD may not be appreciated upon external examination as the face is commonly distorted as a result of ligature suspension and tongue protrusion through the lips and teeth; therefore, it may be attributed to this instead. Additionally, rigor mortis makes mouth opening difficult or impossible, so jaw movement is not easily assessed. The TMJD is not associated with an open-mouth position as it is during life, so clinical diagnostic criteria cannot be employed after death. In relation to the internal examination procedure, the head incision is bicoronal and located retroauricular; thus, it does not expose the TMJ (anterior to external auditory canal). A neck dissection may or may not include a facial subcutaneous dissection, and during this, the masseter and parotid are left in place; thus, the TMJ is not exposed.

PMCT is a well-established and validated tool used in medicolegal death investigation [13] providing a permanent digital record of the deceased and revealing significant findings prior to autopsy. It is a superior technique to autopsy for the demonstration of skeletal trauma [14] and is an invaluable adjunct to autopsy in modern forensic pathology practice. Not all centers have the capacity to integrate PMCT into routine practice, and in those that do, it may be a recent addition to the forensic pathology diagnostic armamentarium. The authors speculate that this may have contributed to the lack of recognition of TMJD before now.

This case report highlights a new observation revealed by PMCT in a commonly encountered type of death—temporomandibular joint dislocation in cases of hanging. Further work is required to elucidate the prevalence of this finding and its characteristics among the population of hanging deaths.

## Data Availability

Not applicable.

## Footnotes

1 This is likely an overestimation of the magnitude of these deaths as they were not reported separately from strangulation.
